# Scalable Production and Multifunctional Coating of Gold Nanostars for Catalytic Applications

**DOI:** 10.3390/nano15090692

**Published:** 2025-05-03

**Authors:** Silvia Nuti, Adrián Fernández-Lodeiro, Inmaculada Ortiz-Gómez, Carlos Lodeiro, Javier Fernández-Lodeiro

**Affiliations:** 1 BIOSCOPE Research Group, LAQV-REQUIMTE, Chemistry Department, NOVA School of Science and Technology (FCT NOVA), Universidade NOVA de Lisboa, 2829-516 Caparica, Portugal or silvia.nuti3@unibo.it (S.N.); cle@fct.unl.pt (C.L.); 2 Department of Electrical and Computer Engineering, University of Cyprus, Nicosia 2112, Cyprus; 3 Department of Physical and Analytical Chemistry, University of Oviedo, E-33006 Oviedo, Spain; ortizinmaculada@uniovi.es; 4 PROTEOMASS Scientific Society, 2825-466 Costa de Caparica, Portugal

**Keywords:** gold, platinum, nanostars, mesoporous silica coating, catalysis

## Abstract

Gold nanostars (AuNSTs) stabilized with adenosine monophosphate (AMP) were synthesized using a scalable method, achieving a 30-fold yield increase compared to previous studies using AMP as a shaping agent, while also reducing the reaction time to 3 h. The AuNSTs were coated with mesoporous silica (mSiO_2_) via a robust approach, producing the AuNSTs@mSiO_2_ nanoparticles (NPs) with tunable thicknesses and consistent optical properties for a range of morphologies. The NPs were additionally coated with platinum (Pt) before synthesizing the mSiO_2_ layer, facilitating a comparative analysis of catalytic activity. The catalytic performance of the bare AuNSTs, the AuNSTs@mSiO_2_, and the AuNSTs@Pt@mSiO_2_ was evaluated through methylene blue reduction, confirming the gold core as the primary catalytic source. The AuNSTs@Pt@mSiO_2_ exhibited enhanced activity, highlighting the potential of the mSiO_2_ coatings. Additionally, solid-phase catalytic tests using 3,3′,5,5′-tetramethylbenzidine (TMB) on cellulose discs demonstrated the effectiveness of these NPs under diverse conditions. These findings showcase the versatility and broad catalytic potential of silica-coated NPs for solution- and solid-phase applications.

## 1. Introduction

Gold nanoparticles (AuNPs) have gained significant attention due to their unique optical, electronic, and catalytic properties, which differ substantially from bulk gold [1]. These versatile nanostructures have found applications in various fields, including catalysis, biomedicine, sensing, and electronics [2]. Advances in synthetic strategies have enabled more precise control over AuNP growth, resulting in a wide range of well-defined morphologies [3].

Recently, the star-shaped gold nanoparticles (AuNSTs) have gained considerable research interest. These branched structures possess unique optical properties, featuring a strong localized surface plasmon resonance (LSPR) band that can be tuned across the 600–1200 nm range [4,5].

Notably, the AuNSTs offer significant electromagnetic field enhancement, even surpassing that of gold nanorods, particularly at the tips of their branched structures [6,7]. However, maintaining these unique properties is challenging due to the migration of low-coordination atoms at the tips to more stabilized regions [8,9], which ultimately leads to an increase in sphericity and alters the optical response of the NSTs. Despite recent advancements, the application of branched gold morphologies remains limited compared to more widely studied shapes, such as nanorods [10].

Recent research has revealed that adenosine monophosphate (AMP) can guide the growth of monodisperse AuNSTs with controlled dimensions and morphology. The AMP adsorbed onto the NSTs surface enables dispersion in organic solvents and imparts vitreophilic properties, facilitating uniform coating with dense silica using Stober’s methodology [4]. Recent studies have highlighted the significant advantages of mesoporous silica-coated AuNSTs compared to their dense silica-coated counterparts. Encapsulating the AuNSTs in a mesoporous silica shell can be beneficial for catalysis, as it provides extra stabilization and protection from heat and the surrounding medium, thus increasing the lifespan and reusability of the NPs [11,12], and can also serve as a molecular reservoir in catalytic applications [13]. The ability to post-modify the AuNSTs with platinum (Pt) enhances their catalytic performance compared to their gold-only counterparts [14], while preserving all the advantages of the mesoporous silica shell.

Additionally, the mesoporous silica shell can also act as a reservoir for loading chemotherapeutic agents; by utilizing the strong near-infrared (NIR) light absorption of the AuNSTs, localized laser heating can be employed to precisely trigger the release of these therapeutic payloads [15]. This release is facilitated by thermally labile components, such as a paraffin sealing layer, which melts under heat, effectively allowing for on-demand drug delivery at the targeted site. Another application may be related to sensing purposes. This strategy is particularly relevant for the development of SERS tags with quantitative capabilities. The silica shell not only prevents plasmonic coupling and uncontrolled aggregation but enhances signal stability and reproducibility, making it ideal for reliable sensing applications [16]. This combination of the AuNSTs and mesoporous silica positions them as versatile platforms for a wide range of applications, from catalysis and drug delivery to highly sensitive SERS-based sensing. Despite the clear advantages of the mesoporous silica-coated AuNSTs in different applications, their practical implementation is often limited by the challenges associated with their synthesis. Some of these methodologies rely on the use of toxic solvents, like DMF [15,17], the use of big quantities of surfactants in the synthesis of the AuNSTs [11], or generally lengthy times for the obtention of the NPs [17]. More importantly, the optical tunability of the coated AuNSTs is typically constrained.

Therefore, a methodology capable of producing large quantities of AuNSTs with different branching degrees, and thus a tunable optical response, while allowing for the tunable coating of mesoporous silica, is highly desirable.

## 2. Results and Discussion

We have optimized the synthesis of AuNSTs assisted by AMP [4] to accelerate production and enable the large-scale fabrication of well-defined nanostars. In addition to the bare AuNSTs, we developed a synthetic methodology for their controlled coating with mesoporous silica. Furthermore, we established conditions for the deposition of platinum onto the AuNSTs surface, followed by a silica coating, to produce the AuNSTs@Pt@mSiO_2_ nanostructures. These materials were subsequently evaluated for their catalytic activity using two model reactions.

### 2.1. Effect of Precursor Concentration on AuNSTs Growth

**Figure 1:**
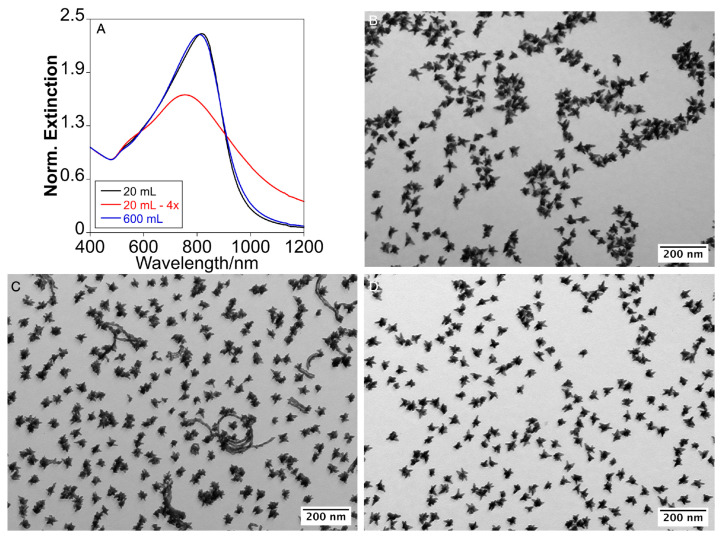
Normalized Extinction spectra (**A**); Transmission Electron Microscopy Images of the AuNSTs obtained by the original protocol (**B**); expansion of the concentration (×4) (**C**); and expansion of volume (×30) (**D**).

Previously, we found that the well-defined AuNSTs could be synthesized at a total gold concentration of 0.3 mM, requiring 24 h at 40 °C to promote the growth of star-shaped NPs [4]. Under these conditions of slow kinetic growth, adjusting the AMP concentration enabled the tuning of the optical response of the resulting AuNSTs. In this study, we selected the conditions to induce the growth of the AuNSTs with a plasmon resonance centered at approximately 811 nm and with an average size of 48.7 ± 7.6 nm, which we used as a model sample (Figure 1A,B and Figure S1A). The size was measured from tip to tip, considering the most distant tips. We investigated possible methods to increase the quantity of the AuNSTs produced per batch, either by raising the total reagent concentration or scaling up the reaction volume.

Initially, we observed that increasing the total reagent concentration under the same conditions led to uncontrolled growth in some NP tips, with a population exhibiting irregular or abnormal tip extension (Figure 1B and Figure S1C,D). Additionally, the remaining NPs displayed a more compact structure with shorter tips compared to those synthesized with the original formulation, resulting in a broader, blue-shifted, and decreased optical response (Figure 1A). Thus, while increasing the reagent concentration enhances the AuNSTs yield, significant variations in morphology and optical response emerged after only a four-fold increase in the precursor concentration compared to the original synthesis.

### 2.2. Scale-Up by Volume Expansion and Temperature Acceleration

Next, we explored expanding the reaction volume as a strategy to maintain the precursor concentrations of the original formulation. Even if scaling by volume expansion is challenging, as previously demonstrated for other gold morphologies, such as nanorods [18], we were able to scale up the reaction volume from 20 mL to 600 mL, a 30-fold increase over the original formulation. To reduce synthesis time, we raised the temperature from 40 °C to 60 °C, which accelerated growth and produced the AuNSTs in just 3 h, as confirmed by the stabilization of their optical response (Figures S1E,F and S2). Interestingly, despite the significantly faster growth kinetics, the morphology of the resulting NPs closely resembled those obtained at 40 °C after 24 h, which validates the role of AMP’s preferential adsorption in directing tip growth, rather than kinetic control, resulting in comparable optical responses in both cases (Figure 1A,D).

Additionally, as happened in the smaller volume reaction, adjusting the AMP concentration enables tuning of the LSPR, providing extra possibilities to the methodology (Figure S3).

### 2.3. The AuNSTs Mesoporous Silica Coating

In order to perform the mesoporous silica coating, and after purification, the AuNSTs were resuspended in 2 mM NaOH [4] to a metallic gold concentration ([Au(0)]) of 0.85 mM, to serve as seeds in the coating experiments. Using cetyltrimethylammonium chloride (CTAC) as a templating agent under basic pH conditions, we induced mesoporous silica growth on the AuNSTs. The CTAC concentration was maintained at 0.8 mM, below the critical micelle concentration (CMC), and NaOH was used to keep the pH between 10.5 and 11, as these conditions have been shown to favor a mesoporous silica coating on Au and Ag NPs stabilized with AMP [19] (see the “Materials and Methods” section in the Supplementary Materials for details).

**Figure 2:**
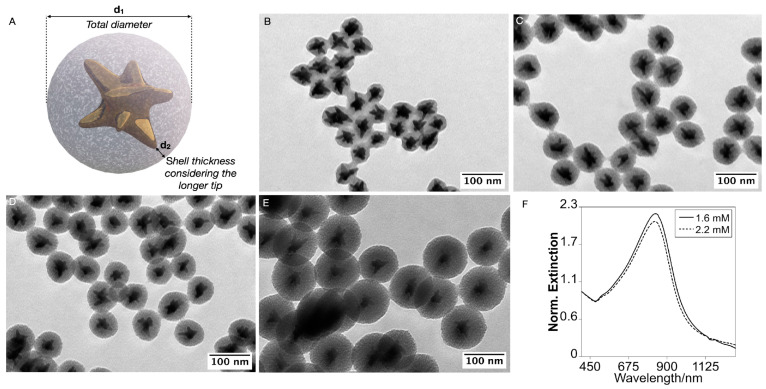
Schematic representation of the characterization of the silica coating (**A**). TEM Images of the AuNSTs@mSiO_2_ obtained using [TEOS] of 1.6 (**B**), 2.2 (**C**), 2.9 (**D**) mM. TEM images of the AuNSTs@mSiO_2_ obtained repeating the coating process with a TEOS concentration of 6.7 mM three times (**E**). Representative extinction spectra of the AuNSTs@mSiO_2_ obtained with 1.6 and 2.2 mM of TEOS (**F**).

Using 5 mL of the AuNSTs at [Au(0)] = 0.85 mM, we found that increasing the concentration of tetraethyl orthosilicate (TEOS) enables precise adjustment of the mesoporous silica coating thickness on the NSTs. To characterize the coating, we defined two parameters: distance 1 (d1), corresponding to the diameter of the core@shell NPs, and distance 2 (d2), which measures the shortest coating thickness along the longest branch of each NST (see Figure 2A for clarity). At a TEOS concentration of 1.6 mM, the resulting NSTs exhibit a thin silica coating with a total size of 55.8 ± 1.2 nm (d1) and a coating thickness of 3.1 ± 1.4 nm, with some tips extending beyond the silica coverage (Figure 2B,F and Figure S4). Increasing the TEOS concentration to 2.2 mM raises the thickness to d1 = 72.8 ± 6.0 nm and d2 = 10.1 ± 2.3 nm (Figure 2C,F and Figure S5), similar to 2.9 mM of TEOS concentration (Figure 2D). Further increasing the TEOS concentration beyond 3 mM does not produce significant dimensional changes (d1 = 78.9 ± 5.6 nm and d2 = 13.8 ± 2.5 nm), but leads to the self-nucleation of the silica nanoparticles, resulting in coreless particles (Figure S6). To increase the coating thickness beyond this limit while minimizing silica self-nucleation, we employed a stepwise growth approach. After performing three consecutive growth steps, the NPs reached a final size of d1 = 111.1 ± 10.4 nm and d2 = 30.1 ± 4.5 nm (Figure 2E and Figure S7).

The optical response of the NSTs remained stable after the coating process. Upon adding TEOS, the primary LSPR experienced a red shift, attributed to the dielectric change in the surrounding medium. This behavior is consistent with observations in gold nanorods [20] and silver nanospheres [19,21]. Additionally, purifying the mesoporous silica-coated nanoparticles through successive washes with ethanol (EtOH), methanol (MeOH), and water induced a blue shift, bringing the optical response of the mesoporous silica-coated NSTs closer to that of the uncoated AuNSTs (Figure S8). This shift primarily results from the substantial removal of the templating agent (CTA⁺) from the silica pores [19,20,21,22]. Similar methodologies typically yield mesopores in the range of approximately 2 to 6 nm, which is sufficient to accommodate small molecules, such as methylene blue and TMB, making it suitable for catalytic applications involving these species [23,24]. Moreover, AuNSTs with different branching degrees can be coated with a mesoporous silica shell applying the same methodology (Figure S9).

### 2.4. Catalytic Reduction of Methylene Blue with the Silica-Coated AuNSTs

In catalysis, the morphology of the nanoparticles plays a crucial role in determining their efficiency. Spherical and star-shaped particles exhibit distinct surface structures, with star-shaped particles possessing more edges and high-energy sites that enhance catalytic activity. Additionally, the atomic arrangement of the different crystallographic planes within these particles significantly influences their reactivity and selectivity, making shape-controlled synthesis a key strategy for optimizing catalytic performance [25]. Taking advantage of the mesoporous structure of the outer silica shell, we investigate here the catalytic potential of these NSTs. Specifically, we explore whether the silica shell’s permeability can permit and modify the catalytic activity of the AuNSTs. To this end, we analyzed the catalytic properties of the different colloids developed in this study using a model reduction reaction, namely, the reduction of methylene blue (MB) (see the “Materials and Methods” section in the Supplementary Materials for details). Control experiments confirm that MB cannot be efficiently reduced by sodium borohydride (NaBH_4_) alone in the absence of metal nanoparticles.

**Figure 3:**
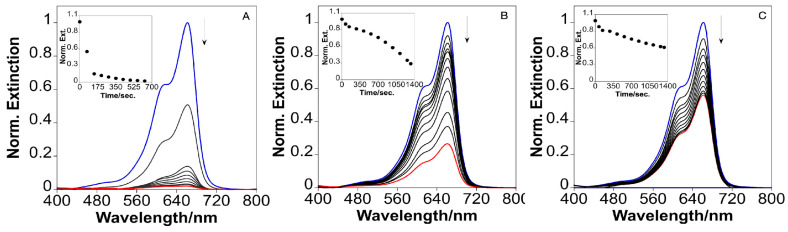
Time-dependent UV-visible spectra for the catalytic reduction of methylene blue by AuNSTs (**A**); and AuNSTs@mSiO_2_ with two different silica thicknesses (**B**,**C**), with the inset showing the plot of time dependent absorbance at 662 nm.

Figure 3 shows the extinction spectra of MB in the presence of bare AuNSTs (A) and the AuNSTs@mSiO_2_ with mesoporous silica shells of increasing thickness (B,C). In all cases, a distinct reduction in MB absorbance intensity is observed over time, indicating the progressive reduction of MB. However, the rate and extent of MB reduction clearly depends on the thickness of the mesoporous silica shell surrounding the Au core. For bare AuNSTs (A), the MB molecules rapidly interact with the Au surface, leading to swift reduction, as evidenced by the significant and rapid decrease in the characteristic MB absorbance peaks. After 10 min, a reduction of approximately 98% is achieved, with an apparent rate constant (*k*_app_) of 0.341 min^−1^. By contrast, when the mesoporous silica shell is added, the reduction kinetics slow down considerably, as shown in panels B and C. This effect becomes more pronounced with increasing shell thickness, with panel B (thinner shell) showing faster MB reduction compared to panel C (thicker shells) (Tables S1 and S2). Specifically, the MB reduction percentages for the AuNSTs@mSiO_2_ with increasing thicker shells were 73.46% and 44.01% after 22 min, with corresponding *k*_app_ values of 0.025 min^−1^ and 0.027 min^−1^. This trend is consistent with a diffusion-limited process, where the MB molecules must permeate through the mesoporous silica shell [26] to reach the metallic core, where electron transfer facilitates reduction [27]. As the silica shell thickens, the diffusion path lengthens, delaying MB access to the Au core and potentially hindering the reduced molecule’s exitfrom the catalyst surface. As a result, the degradation process slows down. However, despite the decrease in the degradation percentage being consistent with the increasing thickness of the mesoporous silica layers, we observe that the *k*_app_ values for the thinnest and thicker silica shells are comparable. This may be due to a combination of factors, including the exposure of some NST tips when the silica shell is thinner (Figure 1B), which could enhance MB reduction. Additionally, the larger silica layer may create a microenvironment that concentrates reactants near the catalyst surface, optimizing diffusion and maintaining a comparable reaction rate. This effect, known as confinement, is well-documented in porous materials and can significantly influence catalytic properties by altering diffusion, phase transformations, and reaction pathways. In such a confined environment, changes in adsorption, geometrical constraints, selective absorption, and modifications to the potential energy surface can enhance reaction selectivity and activity. Furthermore, the mesoporous silica shell can protect the Au surface from aggregation [28].

### 2.5. Platinum and Mesoporous Silica Functionalization of the AuNSTs and Catalytic Applications

To further explore the catalytic role of the metallic core, we coated the AuNSTs with Pt and subsequently encapsulated them in mesoporous silica. This strategy allowed us to directly compare the catalytic performance of the nanostructures while maintaining consistent size and shape, thereby isolating and evaluating the specific contribution of the metal core—gold or platinum—to the overall catalytic activity.

**Figure 4:**
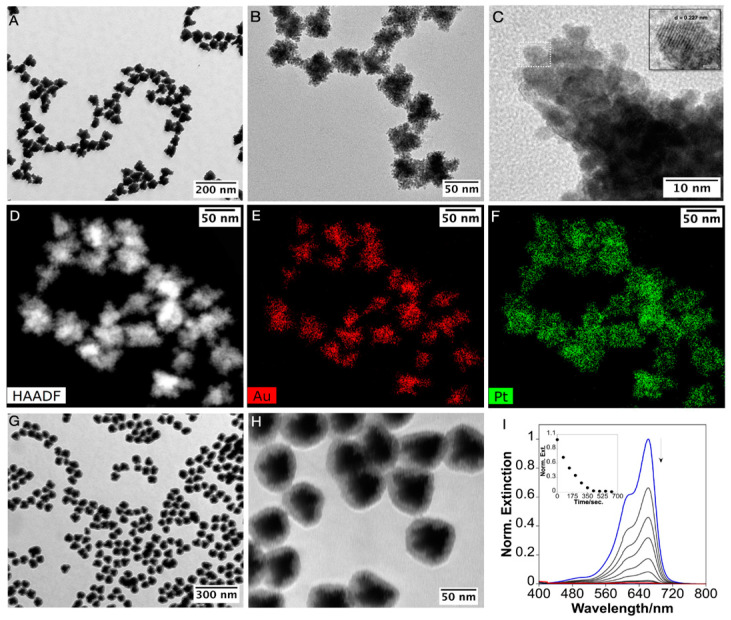
Transmission electron microscopy (TEM) images of the AuNSTs@Pt at different magnifications (**A**,**B**). High-resolution TEM image, with the inset showing a zoomed-in view of the region marked by the dotted box, highlighting an interplanar distance of 0.227 nm corresponding to the Pt(111) plane (**C**). High-angle annular dark-field (HAADF) image (**D**), with EDS elemental maps showing Au in red (**E**) and Pt in green (**F**). TEM images of the AuNSTs@Pt@mSiO_2_ NPs at different magnifications (**G**,**H**). Time-dependent UV-visible spectra for the catalytic reduction of methylene blue by AuNSTs@Pt@SiO_2_NPs, with the inset showing the plot of time dependent absorbance at 662 nm (**I**).

Platinum was deposited onto the AuNSTs by reducing the Pt(II)–AMP complex with ascorbic acid (AA) as a mild reducing agent at 60 °C. (See the “Materials and Methods” section in the Supplementary Materials for details.) Following the synthesis, the NPs exhibited a characteristic black color, accompanied by the disappearance of the LSPR band, indicating the successful deposition of Pt. Electron microscopy images revealed that the AuNSTs were completely covered with a compact and granular Pt shell producing NPs with an average size of approximately 72 nm, with the fully coated branches clearly visible (Figure 4A,B and Figure S10). High-resolution transmission electron microscopy (HR-TEM) images further confirmed that the Pt coating consisted of small Pt crystalline units, with an interplanar distance of 0.227 nm, corresponding to the Pt(111) crystal plane (Figure 4C and Figure S11). This island-like growth mode was consistent with previous observations of Pt-coated branched gold NPs stabilized with polyallylamine [14]. High-angle annular dark-field (HAADF) imaging (Figure 4D) combined with energy dispersive X-ray spectroscopy (EDS), confirmed the bimetallic composition of the NPs, showing a gold-rich core and a platinum-rich shell (Figure 4E,F). Finally, the bimetallic AuNSTs@Pt NPs were coated with mesoporous silica, resulting in uniform AuNSTs@Pt@mSiO_2_ structures without core-free silica NPs, and the silica shell had a thickness of approximately 11 nm (Figure 4G,H and Figure S12).

The absorbance spectra of MB in the presence of AuNSTs@Pt@mSiO_2_ NPs (Figure 4I) demonstrate that, despite the diffusion barrier imposed by the silica, the catalytic reduction of MB remains highly efficient, achieving approximately 99.36% reduction in ~10 min with a *k*_app_ of 0.500 min^−1^. When comparing these results to the AuNSTs@mSiO_2_ with a similar mesoporous silica shell (Figure 3, panel B), it is evident that the presence of the AuNSTs@Pt core significantly enhances the reduction kinetics (Tables S1 and S2). The AuNSTs@Pt@mSiO_2_ NPs exhibit a faster and more complete reduction of MB compared to both their AuNSTs@mSiO_2_ counterparts and bare AuNSTs, suggesting that the catalytic efficiency of the AuPt core is superior even when MB diffusion is similarly constrained by the mesoporous silica. The improved performance of the AuNSTs@Pt@mSiO_2_ NPs likely results from the island-like growth of Pt on the Au core, which enhances catalytic activity through multiple mechanisms. It creates a textured surface, increasing the surface area and providing more catalytic sites. Additionally, the Au–Pt interfaces generate synergistic effects, promoting intermediate oxidation and ion transport. Finally, the partial exposure of the Au surface, along with platinum islands, forms bimetallic catalytic sites [14,29,30]. Notably, the mesoporous silica-coated nanoparticles remain stable and preserve their catalytic performance for at least three months following their synthesis. The silica shell, despite introducing a diffusion barrier, offers substantial stability under a variety of conditions [12,31,32], making the AuNSTs@mSiO_2_ and the AuNSTs@Pt@mSiO_2_ NPs well-suited for applications where durability and resistance to degradation are important.

Finally, the catalytic activity of these NPs was assessed by depositing 3,3′,5,5′-tetramethylbenzidine (TMB) and the NPs onto a cellulose disc.

To this end, the catalytic activity of the AuNSTs, the AuNSTs@SiO_2__1, the AuNSTs@SiO_2__2, and the AuNSTs@Pt@mSiO_2_ was investigated using cellulose discs as the support. This evaluation was performed in triplicate to ensure the reliability and reproducibility of the results, which were consistent across all trials. The findings revealed that the catalytic activity of the AuNSTs, the Au@SiO_2__1, and the Au@SiO_2__2 facilitated the oxidation of TMB on the cellulose disc, as depicted in Figure S13A–C. The presence of the silica coating resulted in a more gradual and controlled catalytic process, with the color change appearing after approximately 12 min. By contrast, the AuNSTs@Pt@SiO_2_ exhibited the highest catalytic activity, rapidly catalyzing the oxidation of TMB and causing an immediate color change to intense blue on the cellulose disc, as shown in Figure S13D. This rapid reaction highlights the enhanced catalytic efficiency provided by the Pt deposition. It can be concluded that, although the blue color for the AuNSTs@SiO_2__1 and the AuNSTs@SiO_2__2 was less intense than that observed for the AuNSTs@Pt@SiO_2_, the catalytic activity was still effective, showing that the silica-coated NPs can mediate a controlled oxidation reaction.

Our research demonstrates that the diffusion of molecules through mesoporous silica layers can be exploited in catalysis. Although the reaction rate decreases, likely due to the time required for the reagents to diffuse, the mesoporosity can be beneficial in challenging catalytic reactions where simpler stabilizers might degrade (e.g., at high temperatures or in extreme acidic conditions). Mesoporous silica, on the other hand, offers increased stability under these harsh conditions [33].

## 3. Conclusions

In this study, we have successfully demonstrated the scalable synthesis of AuNSTs using AMP as a shape-directing agent and capping agent, significantly reducing reaction time through temperature control. We achieved tunable coatings of the AuNSTs with a mesoporous silica layer and a Pt shell, and further demonstrated the feasibility of combining both approaches to produce AuNSTs@Pt@mSiO_2_ NPs. Our experiments on MB reduction confirmed that the silica shell does not hinder the catalytic properties of the NSTs. On the contrary, the silica layer acts as an effective stabilizing barrier, preventing NP aggregation, a common challenge in catalytic processes involving metallic NPs. These findings highlight the potential of mesoporous silica as a protective shell that enhances the stability and longevity of metal-based catalysts in various applications.

Furthermore, our research highlights that molecular diffusion through the silica pores enables the application of these catalysts for both reduction and oxidation reactions. The mesoporous silica shell may also influence molecular access, potentially allowing the smaller molecules to pass through while restricting the larger ones, functioning as a molecular sieve. Future investigations should focus on selective catalysis, using this sieve-like property to catalyze substrates of specific dimensions in the presence of larger, non-reactive molecules, enhancing both catalytic efficiency and substrate specificity in complex mixtures.

## Data Availability

Data are contained within the article and Supplementary Materials.

## Supplementary Materials

The following supporting information can be downloaded at: https://www.mdpi.com/article/10.3390/nano15090692/s1, Table S1: MB reduction % for the different NSTs systems; Table S2: *k*app for the reduction of MB for the different NSTs systems, Figure S1. TEM images of AuNSTs demonstrate scalability in synthesis: AuNSTs synthesized using the original formulation (A,B), synthesis with a 4-fold increase in concentration (C,D), and synthesis with a 30-fold increase in reaction volume (E,F). Figure S2. Extinction spectra showing the growth of NSTs in a 600 mL reaction at 60 °C over time. After 150 min, the absorption at 400 nm reaches 0.713, corresponding to 99% of the reduced Au (A). Experimental setup used for synthesizing large volumes of NSTs. (The concentration of Au(0) corresponding to the absorption at 400 nm was calculated based on previously reported methods^18^) (B)., Figure S3. Normalized extinction spectra of AuNSTs synthesized in a 600 mL reaction volume at different [AMP] concentrations: 0.5 mM (black spectrum), 0.82 mM (blue spectrum), and 0.88 mM (red spectrum)., Figure S4. TEM images of AuNSTs coated with mesoporous silica using 1.6 mM of [TEOS] at different magnifications, Figure S5. TEM images at different magnification of AuNSTs coated with mesoporous silica using 2.2 mM of [TEOS], Figure S6. TEM images at different magnification of AuNSTs coated with mesoporous silica using 3.5 mM of [TEOS]. Note the presence of core free mesoporous silica NPs, Figure S7. TEM images at different magnification of AuNSTs coated with mesoporous silica after 3 steps-growth with 6.7 mM of total [TEOS], Figure S8. Normalized extinction spectra of AuNSTs, AuNSTs@mSiO_2_, and AuNSTs@mSiO_2_ after multiple EtOH, MeOH, and water purifications, Figure S9. TEM images of AuNSTs with different branching degrees coated with mesoporous silica shell, Figure S10. TEM images of AuNSTs@Pt at different magnifications, Figure S11. HR-TEM images of different tips showing Pt crystal structures completely cover the Au surface, Figure S12. TEM images of AuNSTs@Pt@mSiO_2_ at different magnifications, Figure S13. Images of the paper discs after catalytic activity of (A) AuNSTs, (B) AuNSTs@mSiO_2__1, (C) AuNSTs@mSiO2_2, and (D) AuNSTs@Pt@mSiO_2_ in the oxidation of TMB substrate. The analyses were carried out in triplicate. Materials and methods, with the Materials, Synthesis of Au seeds, Synthesis of AuNSTs, Pt coating of AuNSTs, Mesoporous silica coating of AuNSTs and AuNSTs@Pt, Catalytic studies and Characterization. References [18,34,35,36] are cited in the Supplementary Materials.
