# Supplementary material for: Scalable Production and Multifunctional Coating of Gold Nanostars for Catalytic Applications
Source: Nanomaterials (Basel). 2025 May 3;15(9):692. doi: 10.3390/nano15090692 (PMC12074007; doi:10.3390/nano15090692)
Supplement: Supplementary file 1 [file nanomaterials-15-00692-s001.zip › nanomaterials-3571932-supplementary.pdf]

nanomaterials

Supporting information

Scalable Production and Multifunctional Coating of Gold Nanostars for Catalytic Applications

Silvia Nuti [1,†], Adrián Fernández-Lodeiro [2,*], Inmaculada Ortiz-Gómez [3], Carlos Lodeiro [1,4] and Javier Fernández-Lodeiro [1,4,*]

1 BIOSCOPE Research Group, LAQV-REQUIMTE, Chemistry Department, NOVA School of Science and Technology (FCT NOVA), Universidade NOVA de Lisboa, 2829-516 Caparica, Portugal; s.nuti@campus.fct.unl.pt (S.N.); cle@fct.unl.pt (C.L.)
2 Department of Electrical and Computer Engineering, University of Cyprus, Nicosia 2112, Cyprus
3 Department of Physical and Analytical Chemistry, University of Oviedo, E-33006 Oviedo, Spain; ortizinmaculada@uniovi.es
4 PROTEOMASS Scientific Society, 2825-466 Costa de Caparica, Portugal
* Correspondence: fernandez-lodeiro.adrian@fct.unl.pt (A.F.-L.); j.lodeiro@fct.unl.pt (J.F.-L.)
† Current address: Department of Chemistry "Giacomo Ciamician", University of Bologna, Via Gobetti 85, 40129 Bologna, Italy.

Table of Contents:

	Page
**Table S1** MB reduction % for the different NSTs systems	2
**Table S2** k_{app} for the reduction of MB for the different NSTs systems	2
**Figure S1.** TEM images of AuNSTs demonstrate scalability in synthesis: AuNSTs synthesized using the original formulation (A, B), synthesis with a 4-fold increase in concentration (C, D), and synthesis with a 30-fold increase in reaction volume (E, F).	3
**Figure S2.** Extinction spectra showing the growth of NSTs in a 600 mL reaction at 60 °C over time. After 150 minutes, the absorption at 400 nm reaches 0.713, corresponding to 99% of the reduced Au (A). Experimental setup used for synthesizing large volumes of NSTs. (The concentration of Au(0) corresponding to the absorption at 400 nm was calculated based on previously reported methods[18]) (B).	4
**Figure S3.** Normalized extinction spectra of AuNSTs synthesized in a 600 mL reaction volume at different [AMP] concentrations: 0.5 mM (black spectrum), 0.82 mM (blue spectrum), and 0.88 mM (red spectrum).	4
**Figure S4.** TEM images of AuNSTs coated with mesoporous silica using 1.6 mM of [TEOS] at different magnifications.	5
**Figure S5**. TEM images at different magnification of AuNSTs coated with mesoporous silica using 2.2 mM of [TEOS].	5
**Figure S6**. TEM images at different magnification of AuNSTs coated with mesoporous silica using 3.5 mM of [TEOS]. Note the presence of core free mesoporous silica NPs.	5
**Figure S7**. TEM images at different magnification of AuNSTs coated with mesoporous silica after 3 steps-growth with 6.7 mM of total [TEOS].	6
**Figure S8.** Normalized extinction spectra of AuNSTs, AuNSTs@mSiO_2, and AuNSTs@mSiO_2 after multiple EtOH, MeOH, and water purifications.	6
**Figure S9.** TEM images of AuNSTs with different branching degrees coated with mesoporous silica shell.	6
**Figure S10.** TEM images of AuNSTs@Pt at different magnifications.	7
**Figure S11.** HR-TEM images of different tips showing Pt crystal structures completely cover the Au surface.	7
**Figure S12.** TEM images of AuNSTs@Pt@mSiO_2 at different magnifications.	8

Figure S13. Images of the paper discs after catalytic activity of A) AuNSTs, B) AuNSTs@mSiO_2_1, C) AuNSTs@mSiO_2_2, and D) AuNSTs@Pt@mSiO_2 in the oxidation of TMB substrate. The analyses were carried out in triplicate. 8
Materials and methods:
Materials
Synthesis of Au seeds
Synthesis of AuNSTs
Pt coating of AuNSTs 8
Mesoporous silica coating of AuNSTs and AuNSTs@Pt
Catalytic studies
Characterization
References 11

Table S1. MB reduction % for the different NSTs systems.

NSTs	MB reduction %	Time
**AuNSTs**	98.0%	10 mins
**AuNSTs@mSiO_2_1**	73.5%	22 mins
**AuNSTs@mSiO_2_2**	44.0%	22 mins
**AuNSTs@Pt@mSiO_2**	99.3%	10 mins

Table S2. k_{app} for the reduction of MB for the different NSTs systems.

NSTs	k_{app}
**AuNSTs**	0.341 min^{-1}
**AuNSTs@mSiO_2_1**	0.025 min^{-1}
**AuNSTs@mSiO_2_2**	0.027 min^{-1}
**AuNSTs@Pt@mSiO_2**	0.5 min^{-1}

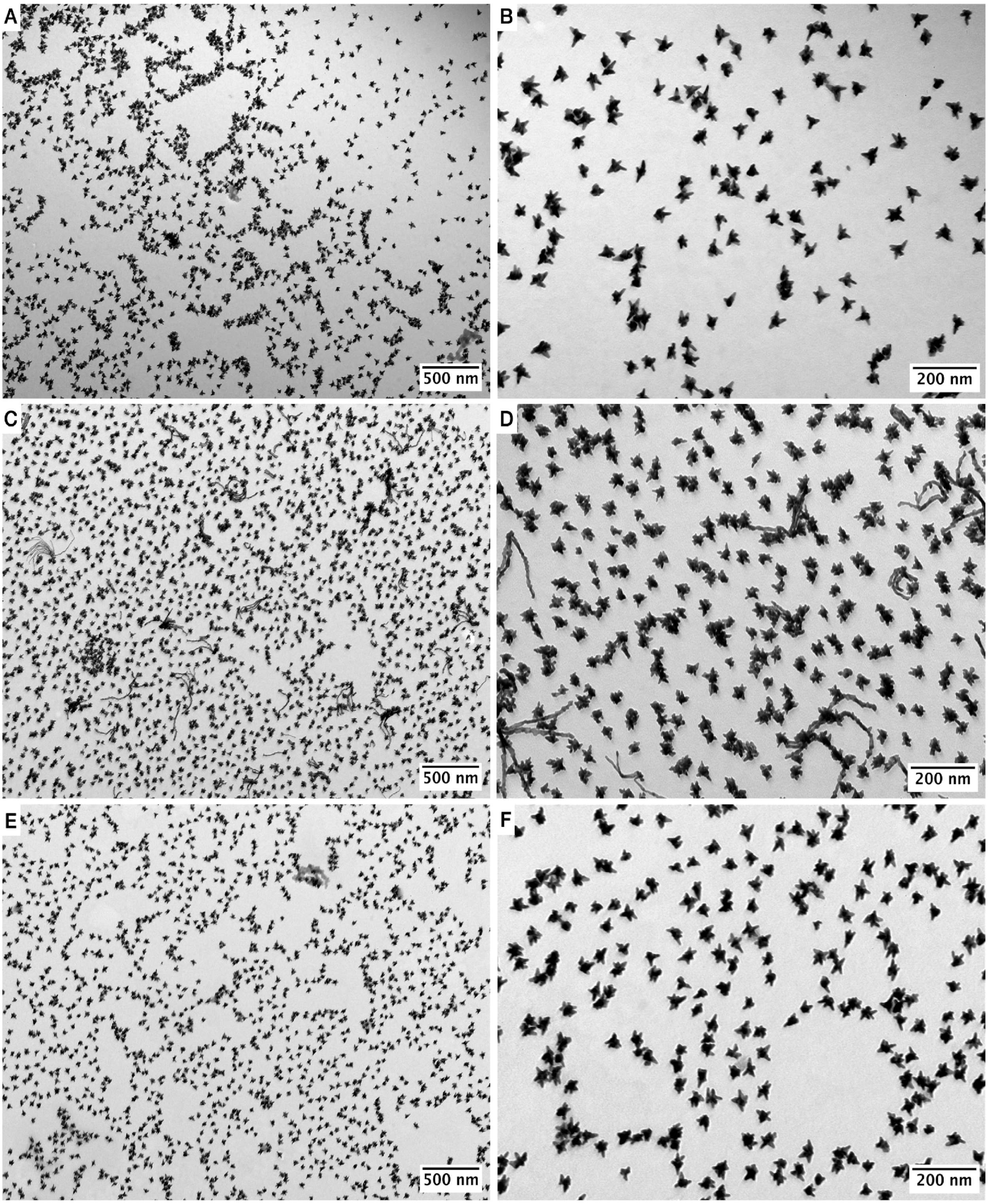

Figure S1. TEM images of AuNSTs demonstrate scalability in synthesis: AuNSTs synthesized using the original formulation (A, B), synthesis with a 4-fold increase in concentration (C, D), and synthesis with a 30-fold increase in reaction volume (E, F).

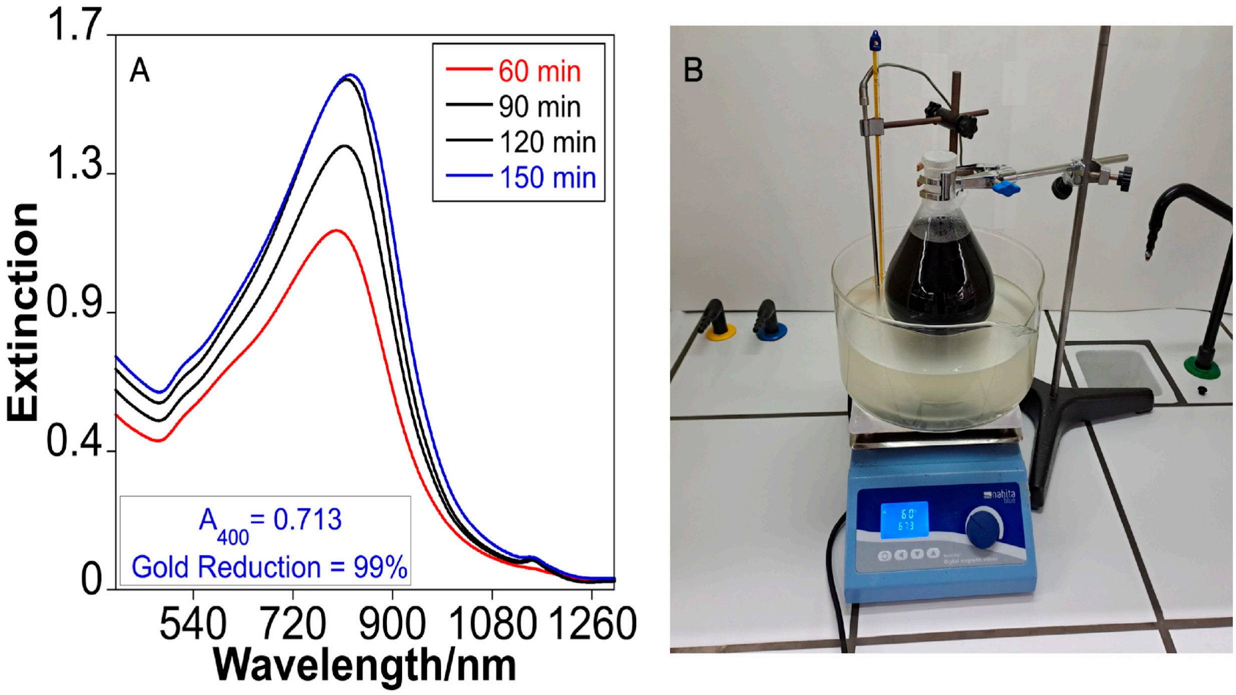

Figure S2. Extinction spectra showing the growth of NSTs in a 600 mL reaction at 60 °C over time. After 150 minutes, the absorption at 400 nm reaches 0.713, corresponding to 99% of the reduced Au (A). Experimental setup used for synthesizing large volumes of NSTs. (The concentration of Au(0) corresponding to the absorption at 400 nm was calculated based on previously reported methods[18]) (B).

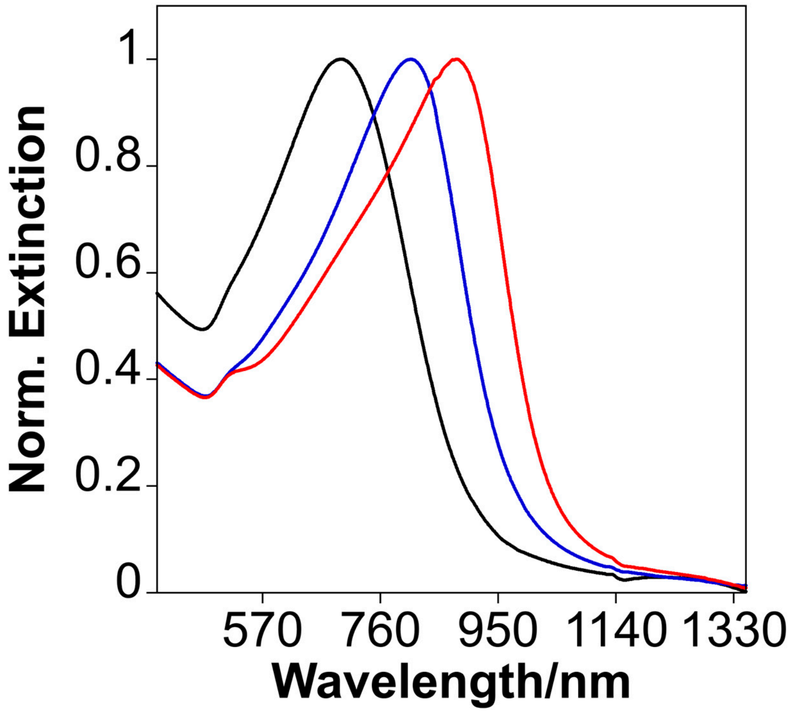

Figure S3. Normalized extinction spectra of AuNSTs synthesized in a 600 mL reaction volume at different [AMP] concentrations: 0.5 mM (black spectrum), 0.82 mM (blue spectrum), and 0.88 mM (red spectrum).

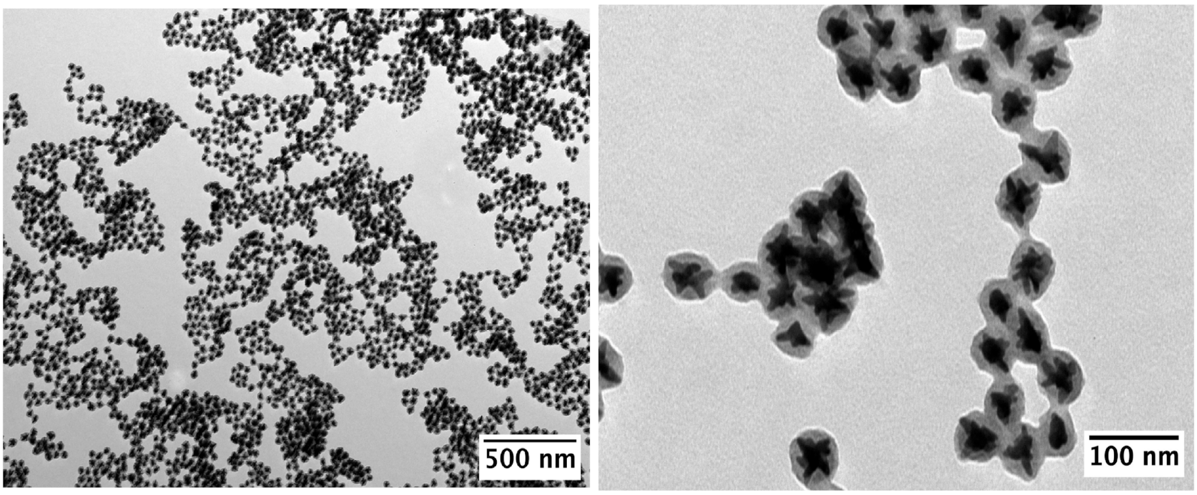

Figure S4. TEM images of AuNSTs coated with mesoporous silica using 1.6 mM of [TEOS] at different magnifications.

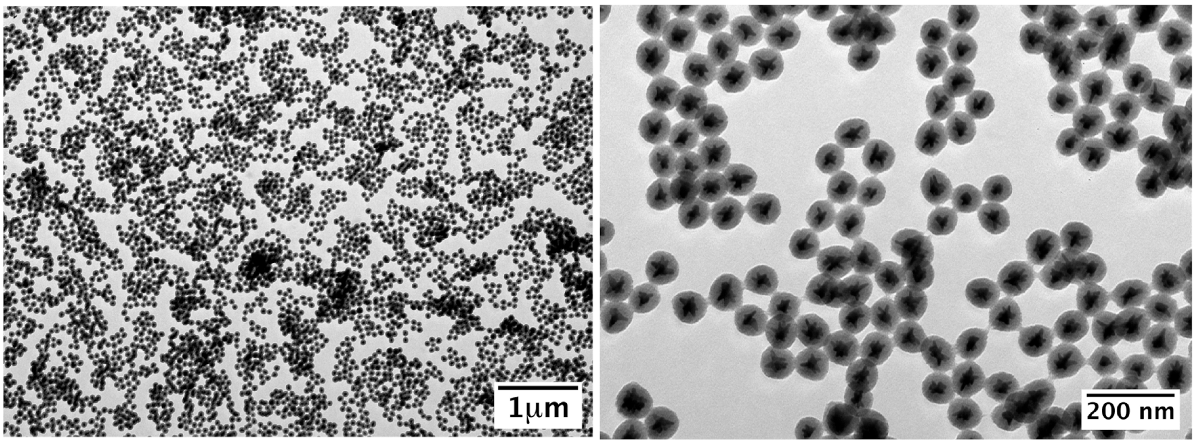

Figure S5. TEM images at different magnification of AuNSTs coated with mesoporous silica using 2.2 mM of [TEOS].

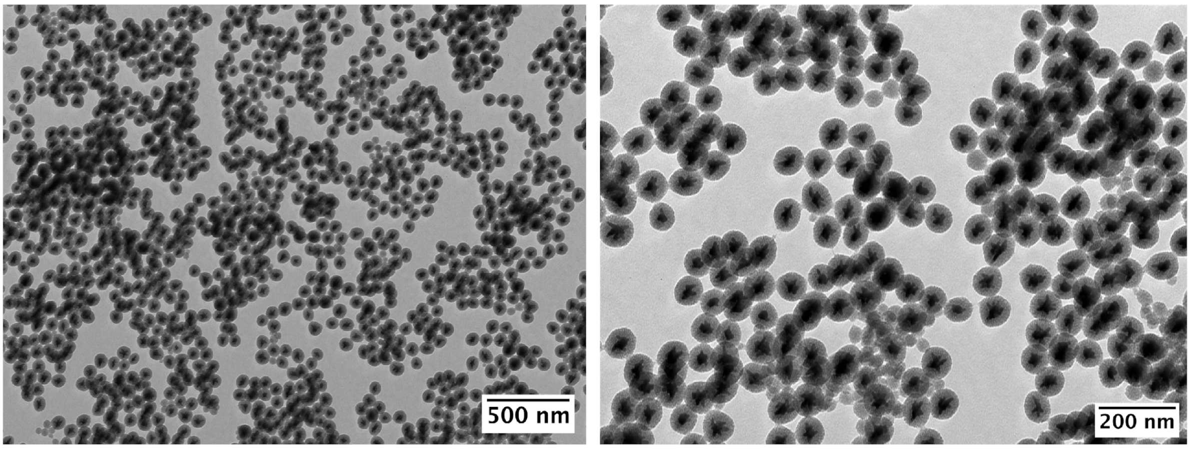

Figure S6. TEM images at different magnification of AuNSTs coated with mesoporous silica using 3.5 mM of [TEOS]. Note the presence of core free mesoporous silica NPs.

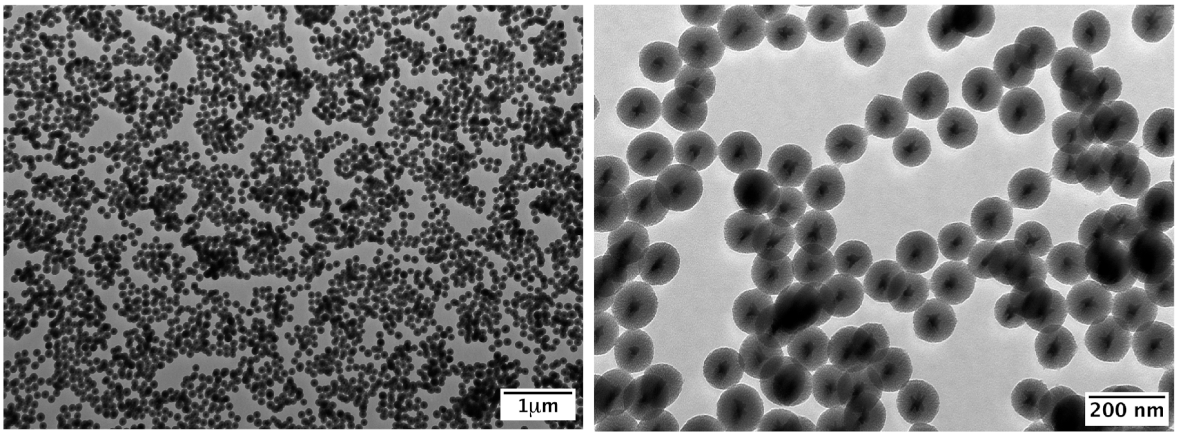

Figure S7. TEM images at different magnification of AuNSTs coated with mesoporous silica after 3 steps-growth with 6.7 mM of total [TEOS].

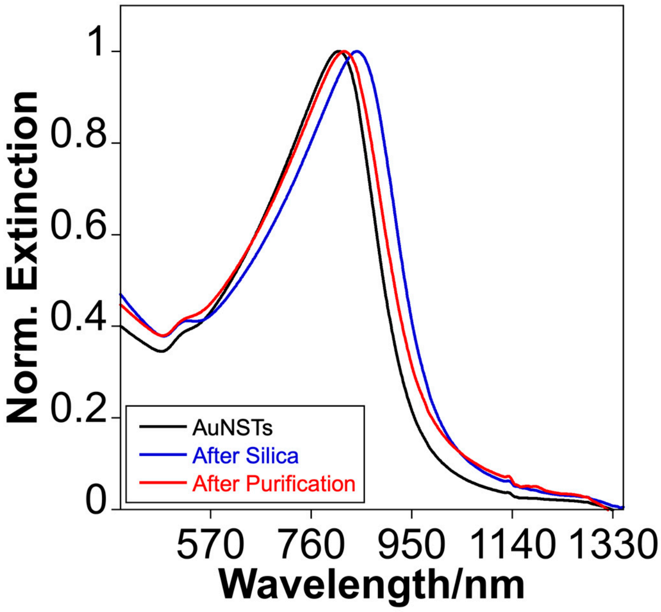

Figure S8. Normalized extinction spectra of AuNSTs, AuNSTs@mSiO_2, and AuNSTs@mSiO_2 after multiple EtOH, MeOH, and water purifications.

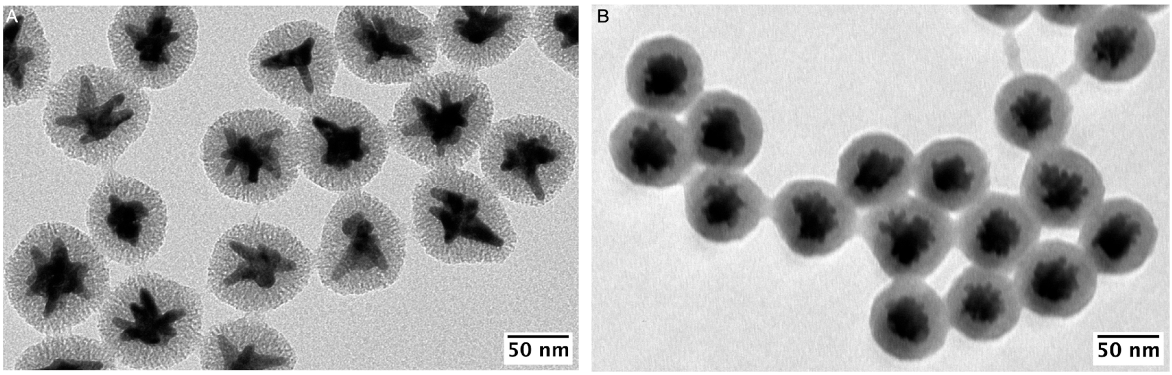

Figure S9. TEM images of AuNSTs with different branching degrees coated with mesoporous silica shell.

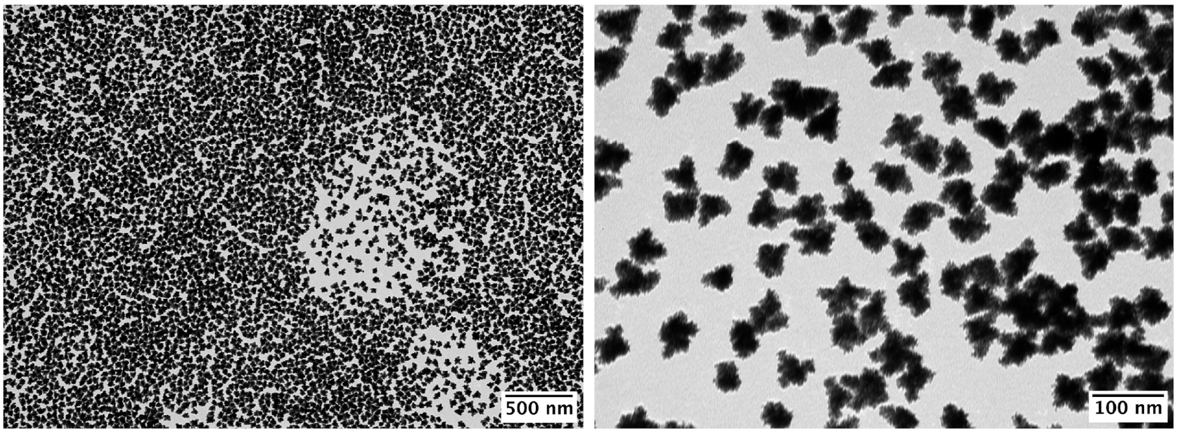

Figure S10. TEM images of AuNSTs@Pt at different magnifications.

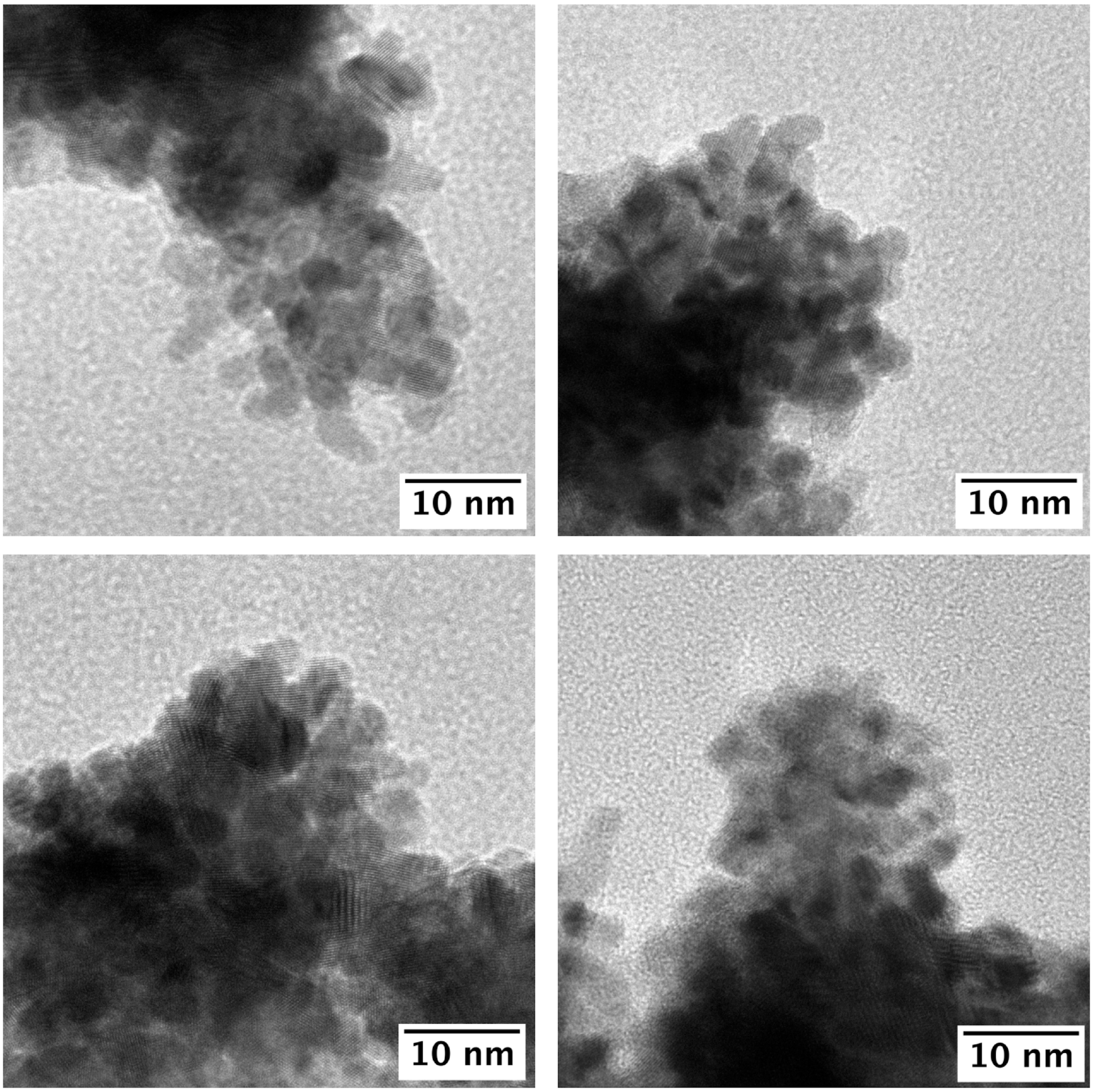

Figure S11. HR-TEM images of different tips showing Pt crystal structures completely cover the Au surface.

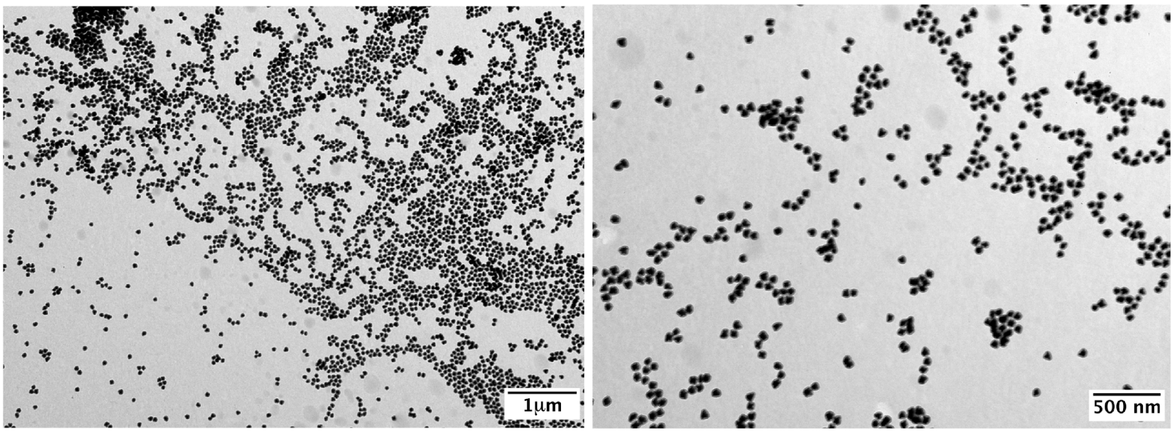

Figure S12. TEM images of AuNSTs@Pt@mSiO$_2$ at different magnifications.

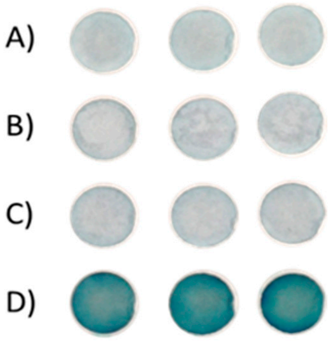

Figure S13. Images of the paper discs after catalytic activity of A) AuNSTs, B) AuNSTs@mSiO$_2$_1, C) AuNSTs@mSiO$_2$_2, and D) AuNSTs@Pt@mSiO$_2$ in the oxidation of TMB substrate. The analyses were carried out in triplicate.

Materials and methods

Materials

Trisodium citrate dihydrate ($C_6H_5Na_3O_7{\cdot}2H_2O$, ≥ 99.5%), Sodium borohydride ($NaBH_4$, ReagentPlus® 99%), L-ascorbic acid (AA) ($C_6H_8O_6$, BioXtra ≥ 99.0% crystalline), Sodium hydroxide (NaOH, BioXtra ≥ 98%, pellets, anhydrous), absolute ethanol (EtOH, ≥ 99.9%) and potassium tetrachloroplatinate (K_2PtCl_4,98%), O-[2-(3-mercaptopropionylamino)ethyl]-O′-methylpolyethylene glycol (PEG-SH, MW 5.000) were obtained from Sigma-Aldrich (Saint Louis, MO, USA). Hexadecyltrimethylammonium Chloride ($C_{19}H_{42}ClN$, 95%) was obtained from TCI Chemicals (Tokio, Japan). Gold(III) chloride trihydrate ($HAuCl_4{\cdot}3H_2O$, 99.9%), Tetraethyl orthosilicate (TEOS)($C_8H_{20}O_4Si$ +99%), and Adenosine-5′-monophosphate disodium salt (AMP) ($C_{10}H_{12}N_5Na_2O_7P$, ≥98.0%) were obtained from Alfa Aesar (Ward Hill, MA, USA). Methylene blue (MB) ($C_{16}H_{18}ClN_3S \cdot 3H_2O$, ≥95%) ,ELISA substrate solutions: 3,3′,5,5′-tetramethylbenzidine (TMB) ($C_{16}H_{20}N_2$, 0.4g/L), and hydrogen peroxide (H_2O_2), were obtained from Thermo Fisher Scientific (Waltham, MA, USA). Quantitative filter paper (reference 1238, basis weight 85 gr, thickness 200 μm; retention 20-25 μm) from Filtros Anoia. S.A. (Barcelona, Spain).

All reagents were used as received without further purification.

Ultrapure water (type I) was used for the preparation of all the water-based solutions. The glassware was cleaned with aqua regia before the experiments

Synthesis of Au seeds

The synthesis of Au seeds was adapted from a previously reported method with minor modifications. In a round-bottom flask at room temperature (RT), 20 mL of ultrapure water containing 0.125 mM $HAuCl_4$ and 0.25 mM trisodium citrate were combined under vigorous stirring. Subsequently, 300 μL of freshly prepared 0.01 M $NaBH_4$ was rapidly injected into the solution. Stirring was then reduced, and the reaction was allowed to proceed for an additional 15 minutes. The solution was subsequently heated to 40 °C with agitation for 60 minutes. The resulting seeds were used directly without further dilution.

Synthesis of AuNSTs

In a 1 L round bottom flask containing 548.4 mL of water, 9 mL of $HAuCl_4$ (20 mM) and 24.9 mL of AMP (20 mM) were added under magnetic stirring. After 5 minutes of stirring, 15 mL of ascorbic acid (20 mM) and 2.7 mL of the seed solution were introduced. The stirring was maintained for an additional 5 minutes, after which the flask was placed in a water bath at 60 °C for 3 hours.

Following the synthesis, the colloidal solution was centrifuged at 6500 rpm for 30 minutes, repeated twice, using 2 mM NaOH as the dispersing medium. The AuNSTs were then redispersed in approximately 150 mL of 2 mM NaOH. The concentration of the colloidal solution was subsequently adjusted to [Au(0)] = 0.85 mM, based on the absorbance at 400 nm.[18] This solution was stored at ~5 °C and demonstrated consistent performance in coating experiments for a minimum of two weeks.

Pt coating of AuNSTs

AuNSTs (7.06 mL, [Au(0)] = 0.85 mM) were centrifuged and redispersed in 20 mL of Milli-Q water. The colloid was transferred to a round-bottom flask, and the following were added under stirring: 400 μL of 0.2 M NaOH. After one minute, 150 μL of a 20 mM K_2PtCl_4 solution was introduced, followed by 600 μL of 20 mM ascorbic acid (AA) after 30 seconds. After 5 minutes of stirring, the additions of K_2PtCl_4 and AA were repeated.

The reaction mixture was stirred for an additional 5 minutes, and the flask was then immersed in a thermostated oil bath at 60 °C under continuous stirring. After 3 hours, 1 mL of 20 mM AMP was added, and the reaction was stirred for an additional hour at room temperature (RT).

The resulting colloidal solution was centrifuged three times using 2 mM NaOH as the dispersing medium and was finally redispersed in 5 mL of 2 mM NaOH for subsequent mesoporous silica coating.

Mesoporous silica coating of AuNSTs and AuNSTs@Pt

A total of 24.32 mL of water and 480 μL of 50 mM CTAC were added to a round-bottom flask. Subsequently, 5 mL of NPs in 2 mM NaOH (AuNSTs or AuNSTs@Pt) were introduced dropwise into the reaction mixture under vigorous magnetic stirring. After 5 minutes, 200 μL of 0.1 M NaOH were added, and the solution was moderated stirred for 4 hours.

Following this, 1 mL of ethanol containing TEOS (between 11–40 μL, see main text) was added dropwise in 200 μL increments every 10 minutes under stirring. The flask was then sealed, and slow stirring was maintained for 24 hours at room temperature (RT).

The resulting AuNSTs@mSiO_2 and AuNSTs@Pt were purified via three centrifugation cycles (5000 rpm, 30 minutes each) using ethanol as the washing medium for TEM analysis.

The colloidal solutions were stored in ethanol at 5 °C for further use. The silica concentrations discussed in the main text were calculated based on a total volume of 30 mL, excluding the 1 mL of added ethanol.

The multi-step coating process was carried out in three consecutive steps, each using 15 μL of TEOS. Between each step, the nanoparticles were centrifuged and resuspended in 29.8 mL of 0.8 mM CTAC. For each step, 300 μL of 0.1 M NaOH was added to the suspension, which was moderately stirred for 90 min. Subsequently, 1 mL of ethanol containing 15 μL of TEOS was added dropwise in 200 μL increments every 10 minutes under stirring. This process was repeated a total of three times.

Catalytic studies

The colloids were purified through successive centrifugation cycles to prepare them for catalytic applications. Initially, two cycles were performed using methanol, followed by additional cycles with water. Between each centrifugation step, the colloids were subjected to an ultrasonic bath at 40°C for 10 minutes to enhance the removal of the CTAC template.

Reduction of MB in liquid

In a quartz cuvette were added 1865 μL of water, 30 μL of MB 1mM and 100 μL of $NaBH_4$ 0.2 M after acquiring the first UV-Vis spectrum, 5 μL of NPs solution with a $[Au^0]$= 0.33 mM (calculated according to the Abs at 400nm of the NPs solution [18]) were added. Afterwards series of UV-spectra were acquired in between 300 and 800 nm for a total of 10 or 20 spectra.

Calculation of the percentage of MB reduction over time and k_{app}

The percentage of MB reduction at each time point was calculated using the following formula[34]:

$$Reduction\ \% = \frac{A_0 - A_t}{A_0} \times 100$$

Where A_0 is the initial absorbance of MB and A_t is the absorbance at time t. By measuring the absorbance (A) of absorption peak of MB at 662 nm with reaction time and plotting $\ln(A_0/A_t)$ versus reaction time (t), the apparent reaction rate constant (k_{app}) was calculated.[35]

TMB paper disks method

To assess the catalytic activity of the nanoparticles in the solid phase, 10 μL of TMB 1.6 mM was added to 8 mm diameter cellulose discs. The discs were then allowed to dry for 5 minutes, after which another 10 μL nanoparticles were added to the paper disc. Later, 10 μL of hydrogen peroxide 30% was dropped into the disc, and the appearance of blue color on the disc was monitored as a result of the TMB oxidation reaction catalyzed by the nanoparticles. The color change was visually monitored, and the paper discs were photographed with a digital camera and using a lightbox to maintain all the image-gathering conditions constant for evaluation of color change.

The cellulose discs were imaged with a digital camera (Canon EOS R10, Tokio, Japan) after a catalyzed reaction. The setting conditions used to acquire the picture of the cellulose discs were: ISO 125, shutter speed 1/60 s, aperture value f/8, focal length of 24 mm, automatic white balance, and resolution 4000x6000.

Characterization

The extinction spectra were recorded using a JASCO 770 UV–Vis-NIR and 650 UV-Vis spectrophotometers provided by the PROTEOMASS-BIOSCOPE facility (Caparica,

Portugal). All spectra were recorded using a HELMA 1 cm light path quartz cell. ζ-potential analyses were carried out in a Malvern ZS instrument at 22 °C provided by the PROTEOMASS-BIOSCOPE facility (Caparica, Portugal). Low-magnification transmission electron microscopy (TEM) images were obtained using a JEOL JEM 1010 TEM microscope (JEOL, Tokyo, Japan), working at 100 kV (CACTI Uvigo and University of Porto**).** AuNSTs and AuNSTs@PtNPs selected for TEM size measurements and morphological assessment were functionalized with PEG-SH prior to deposition for low-resolution TEM analysis to enhance dispersion and ensure accurate evaluation. High Resolution Transmission Electron Microscopy (HRTEM) High-Angle Annular Dark-Field Scanning Transmission Electron Microscopy (HAADF-STEM) and Energy Dispersive X-ray Spectrometry (EDX), analyses were provided by the INL facility in Braga, Portugal. Catalysis Data were treated using *Spectragryph software* (F. Menges "Spectragryph - optical spectroscopy software", Version 1.2.16.1, 2022, Germany http://www.effemm2.de/spectragryph/)[36]

References

(18) Scarabelli, L.; Sánchez-Iglesias, A.; Pérez-Juste, J.; Liz-Marzán, L. M. A "Tips and Tricks" Practical Guide to the Synthesis of Gold Nanorods. *J. Phys. Chem. Lett.* **2015**, *6* (21), 4270–4279. https://doi.org/10.1021/acs.jpclett.5b02123.

(34) Kalaycıoğlu, Z.; Özuğur Uysal, B.; Pekcan, Ö.; Erim, F. B. Efficient Photocatalytic Degradation of Methylene Blue Dye from Aqueous Solution with Cerium Oxide Nanoparticles and Graphene Oxide-Doped Polyacrylamide. *ACS Omega* **2023**, *8* (14), 13004–13015. https://doi.org/10.1021/acsomega.3c00198.

(35) Piella, J., Merkoçi, F., Genç, A., Arbiol, J., Bastús, N. G., & Puntes, V. Probing the surface reactivity of nanocrystals by the catalytic degradation of organic dyes: the effect of size, surface chemistry, and composition *J. Mater. Chem. A*, **2017**,5, 11917-11929

(36) Menges, F. Spectragryph - Optical Spectroscopy Software. **2022**, *Version 1.* (http://www.effemm2.de/spectragryph/).
